# Supplementary material for: Forecasting the outcome of spintronic experiments with Neural Ordinary Differential Equations
Source: Nat Commun. 2022 Feb 23;13:1016. doi: 10.1038/s41467-022-28571-7 (PMC8866480; doi:10.1038/s41467-022-28571-7)
Supplement: Supplementary file 1 — Supplementary Information [file 41467_2022_28571_MOESM1_ESM.pdf]

Supplementary Information: Forecasting the outcome of spintronic experiments with Neural Ordinary Differential Equations

Xing Chen[1,2], Flavio Abreu Araujo[3,4], Mathieu Riou[4], Jacob Torrejon[4], Dafiné Ravelosona[2], Wang Kang[1], Weisheng Zhao[1], Julie Grollier[4], and Damien Querlioz[2*]

[1]Fert Beijing Institute, MIIT Key Laboratory of Spintronics, School of Integrated Circuit Science and Engineering, Beihang University, Beijing 100191, China.
[2]Université Paris-Saclay, CNRS, Centre de Nanosciences et de Nanotechnologies, Palaiseau, France.
[3]Institute of Condensed Matter and Nanosciences, Université catholique de Louvain, Place Croix du Sud 1, Louvain-la-Neuve, 1348, Belgium
[4]Unité Mixte de Physique, CNRS, Thales, Université Paris-Saclay, Palaiseau, France.
[*]email: damien.querlioz@c2n.upsaclay.fr

Supplementary Note 1: Training performance for the skyrmion systems, using voltage as input

This note provides additional results on the training process of Neural ODEs on the one-skyrmion system without grain inhomogeneity and the multi-skyrmions system with grain inhomogeneity, using voltage as input. Fig. S1a shows the normalized random sine voltage input used in the training process. Figs. S1b-c show the predicted training output of Δm_z, by Neural ODE (orange dashed curve) and micromagnetic simulation output (blue curve), for the one-skyrmion and the multi-skyrmions system, respectively. Excellent agreement is seen.

Fig. S2 shows the training loss (MSE) of the one-skyrmion system without grain inhomogeneity as a function of iterations, for:

- **a** different numbers of units N_h in hidden layers. We observe that, in general, the greater N_h is, the faster the training loss converges.
- **b** different sampling intervals Δt of the initially observed trajectory y_1 (the unit p is equal to 2.5 ps, as in the main body text). These results show that an accurate Neural ODE model can be trained even if the original continuous time series is downsampled, until a certain point in which the neighboring states of the downsampled trajectory lose correlations.
- **c** different dimensions k of the Neural ODE (with $k-1$ being the number of delays). The Neural ODE model can be successfully trained as long as $k \geq 2$. Generally, the higher the dimension is, the longer time it takes for the model to train.
- **d** different choices of training optimization algorithms. It is shown that the ADAM and stochastic gradient descent (SGD) methods ensure a quick convergence of the loss function.

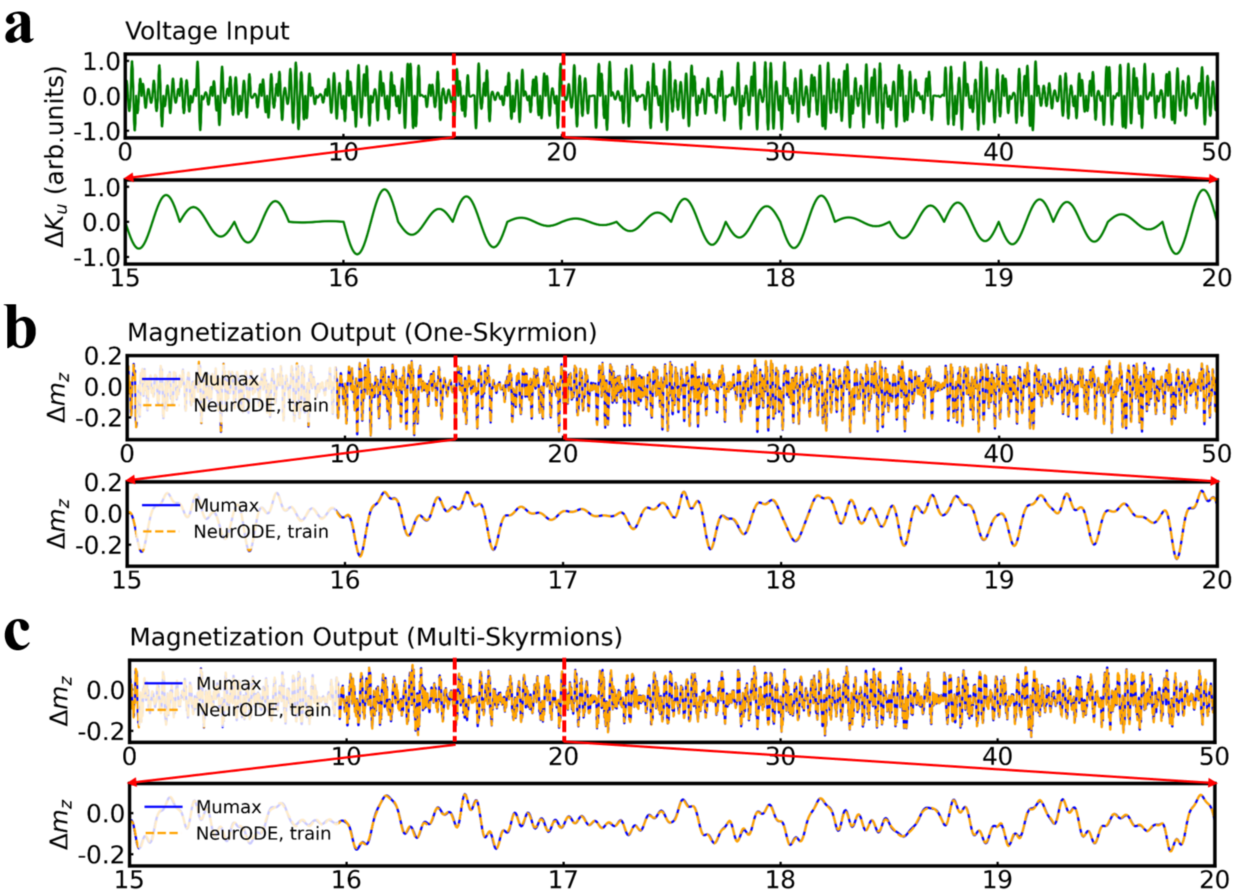

Supplementary Figure 1. Training performance of Neural ODEs for the skyrmion systems using voltage as input. a Normalized random sine voltage input, in the form of ΔK_u, with amplitude ranging from -2$\times 10^5$J/m^3 to 2 $\times 10^5$J/m^3 (corresponding to -1 to 1 in the graph). Here, the variation of the PMA (ΔK_u) is linearly dependent on the voltage applied, because of the VCMA effect. Comparisons of the predicted training output of Δm_z by Neural ODE (orange dashed curve) and micromagnetic simulation output (blue curve) for the one-skyrmion system without grain inhomogeneity (**b**) and the multi-skyrmions system with grain inhomogeneity in (**c**).

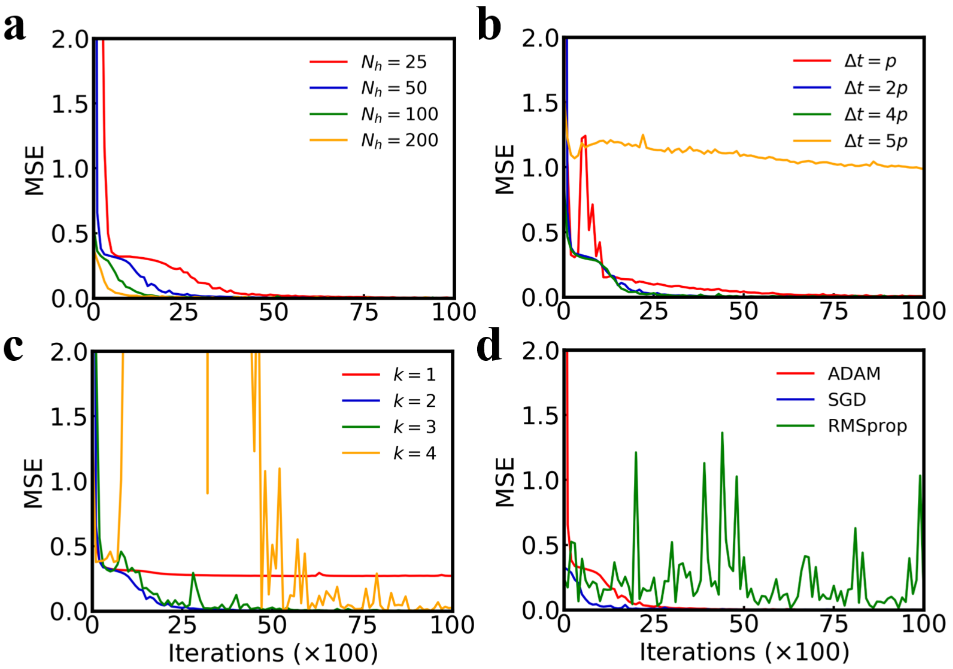

Supplementary Figure 2. Training performance of Neural ODE. Training loss (MSE) of the one-skyrmion system without grain inhomogeneity as a function of iterations, for different numbers of units N_h in hidden layers (**a**), sampling intervals Δt of the initially observed trajectory y_1 (**b**), dimensions of the Neural ODE k (with $k-1$ being the number of delays) (**c**), and optimization algorithms of training (**d**).

Supplementary Note 2: Training Neural ODE models using incomplete noisy data of skyrmion dynamics

This note investigates the training of Neural ODEs over noisy data. The strategy for treating noisy dynamical system comes from the idea, present in the time-delay embedding for state space reconstruction, that increasing the dimension of the reconstructed space can typically decrease the distortion of noise distribution and reduce the amplification of noise level when the delay embedding is transformed to the original state space[1]. Normally, the prediction errors depend on both the noise amplification and the estimation error, which depends on the method of approximation, i.e., the model itself. For most good approximation schemes, the estimation error can be close to zero in the limit of a large number of data points. The prediction errors in this limit are entirely due to the noise. Therefore, by increasing the dimension of the delay vector, a more precise model can be learned.

We use the simulation data of the one-skyrmion system as a demonstration. We obtain noisy training data by artificially adding random Gaussian noise with mean $\mu = 0$ and standard deviation $\sigma_{noi} = 0.1, 0.5$ and 1 into the normalized training data (Δm_z), as shown in Fig. S3a. Fig. S3c presents the training loss (MSE) as a function of iterations, obtained when training Neural ODEs of dimension $k = 2, 4, 8, 12$ over these noisy data with different standard deviations. These results differ from the training results obtained in the noiseless system (see Fig. S2c), where $k \geq 2$ guarantees a sufficiently good model to be trained. Here, the larger the k, the better the model is. On the other hand, training Neural ODE with a large k value (e.g., for k = 12) does not always ensure stable convergence, as seen in the low noise system, because the delayed system dynamics becomes a stiff problem to be solved in the high dimensional space, therefore, it is necessary to choose an appropriate k for training. Further, we remark that the fitted standard deviations of the training error distribution σ_{err} of the trained Neural ODEs, compared to

the true trajectories (without noise), gradually approach the standard deviation of the added noise σ_{noi} when k is increased (see Fig. S3b for $\sigma_{noi} = 0.1$). Fig. S3d shows the testing results of the Neural ODE for $k = 2$ and $k = 12$ under different standard deviations of the added noise. Here, the Mean Square Error (MSE) showed in the title of each graph in Fig. S3d is the MSE between the testing trajectory and the true trajectory without noise. The MSE approaches zero, which justifies a good training system, as k increases especially for the low noise system (e.g., for $\sigma_{noi} = 0.1$). However, as the noise level grows, increasing the k value still helps in making more precise prediction with smaller MSE value, it becomes more difficult to lower the error to zero compared to the true trajectory even with a relatively large k value. A total number of data points $n = 15,000$ are used for training and testing, respectively. The Neural ODEs used in this note featured $N_h = 50$ units per hidden layer.

Still, the effect of noise is very complicated in many real world applications, the demonstration presented here provides a perspective of the possibility of making better prediction when only limited information of the noise is known. There are still many other ways to help distinguish the real system dynamics from the dynamics contaminated with noise, for example, by conjoining different filters with the reconstruction scheme.

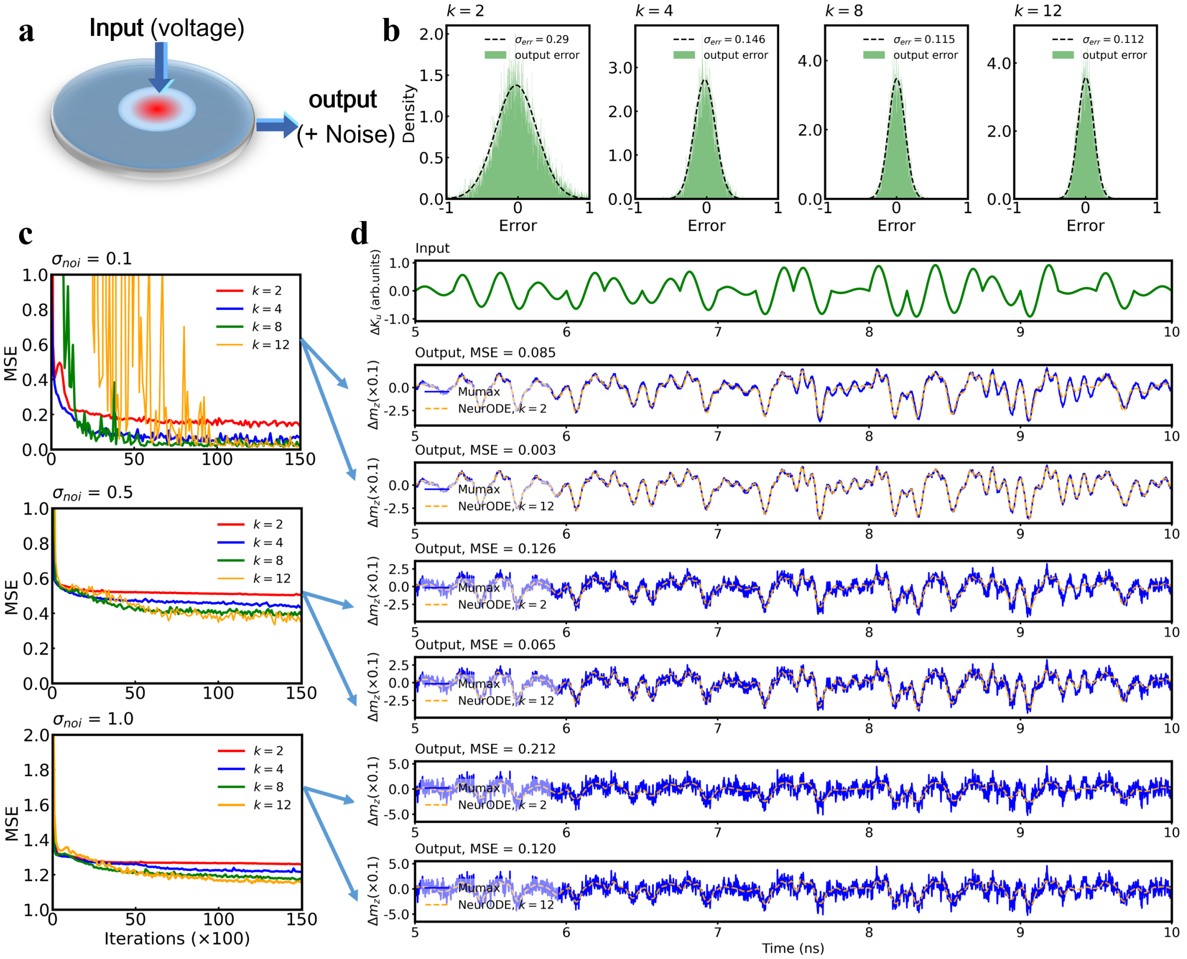

Supplementary Figure 3. Training performance of Neural ODE model using incomplete noisy data. **a** Schematic graph of the procedure to obtain training time series with noise, by artificially adding random values drawn from a Gaussian distribution with mean $\mu = 0$ and standard variance $\sigma_{noi} = 0.1, 0.5$ and 1 respectively into the output time series of the normalized Δm_z. **b** Training error distribution of the trained Neural ODE compared to the true trajectory without noise for different k values where $\sigma_{noi} = 0.1$. **c** Training loss (MSE) as a function of iterations for $\sigma_{noi} = 0.1, 0.5$ and 1, respectively. **d** Final predicted testing results of Neural ODE for $k = 2$ and $k = 12$ under different standard deviation of the added noise. The Mean Square Error (MSE) showed in the title of each graph is the MSE between the testing trajectory and the true trajectory without noise.

Supplementary Note 3: Comparisons between the time-delayed method and the time-derivative method

This note compares the training performance of the time-delayed method and the time-derivative method to model the Neural ODE. The training data $y(t)$ is taken from the one-skyrmion system as a demonstration. We consider the observed data $s(t)$ as a superposition of a noise $n(t)$ and the clean data $y(t)$, so that $s(t) = y(t) + n(t)$. The noise is assumed to be generated from a Gaussian distribution with zero mean and standard deviation $\sigma_{noi} = 0.5$. We adopt the information at three continuous time points of $(t - \Delta t, t, t + \Delta t)$. For the time-delay method, we consider a vector of variables $\mathbf{s}(t) = (s(t - \Delta t), s(t), s(t + \Delta t))$. For the time-derivative method, the vector $\mathbf{s}(t) = (s(t), \dot{s}(t))$ with $\dot{s}(t) = (s(t + \Delta t) - s(t - \Delta t))/(2\Delta t))$ is employed as the training variables. The variance of the signal $y(t)$ extracted from simulations is $\sigma_{noi}^2 = E[y(t)^2] - E[y(t)]^2 = 1.22$; the noise-to-signal ratio is therefore $\sigma_{noi}/\sigma_{sig} = 0.45$. By contrast, if we consider the real signal derivative as $\dot{y}(t) = (y(t + \Delta t) - y(t - \Delta t))/(2\Delta t))$, the noise-to-signal

ratio of the first order derivative $\dot{s}(t)$) is $\sigma_{noi,der}/\sigma_{sig,der} = 3.22$, which is much larger than that of the original signal $s(t)$.

Figs. S4a and b show the training loss (mean square error, MSE) as a function of iterations for $\sigma_{noi} = 0$ (without noise) and $\sigma_{noi} = 0.5$, respectively. It is observed that both methods show similar training performance w.r.t accuracy when noise is absent from the system, however, the time derivative method performs worse than the time delay method when noise exists. This is reasonable because the noise is amplified during the calculation of the first-order derivative, and thus the convergence becomes difficult.

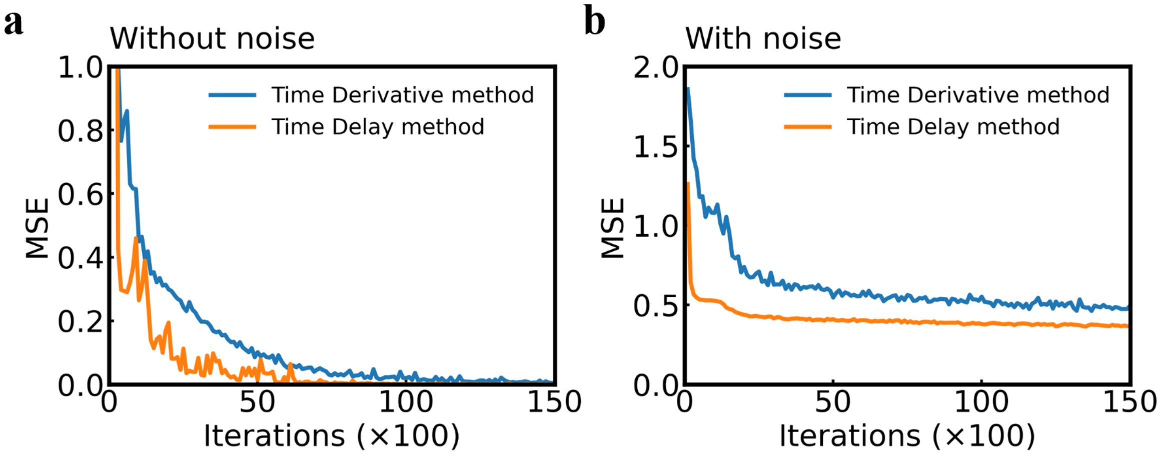

Supplementary Figure 4. Comparison between the time-delay and time-derivative methods in terms of training performance. a Training loss (mean square error, MSE) as a function of iterations for **a** $\sigma_{noi} = 0$(without noise) and **b** $\sigma_{noi} = 0.5$ (with noise), for the one-skyrmion system.

Supplementary Note 4: Mackey-Glass time-series prediction task evaluation

This note provides additional results on the skyrmion-based Mackey-Glass time series prediction task. To perform this task, we send the preprocessed input data into the reservoir, simulated with the trained Neural ODE or Mumax (as a control). With $N_r = 50$ virtual nodes in the reservoir, the total number of input values, including both training and testing set is $50 \times 10,000$. Each value is fed into the reservoir for a duration of $t_{step} = 2p$ ($p = 2.5$ ps). The output trajectory comparison is shown in Fig. S5a and b for the one-skyrmion system without grain inhomogeneity and multi-skyrmions system with grain inhomogeneity, respectively.

Using the trained model, we evaluated the reservoir performance with different N_r values, as shown in Fig. S5c. These results show that the prediction capability of the reservoir is not improved by increasing the number of virtual nodes of the reservoir. On the other hand, Fig. S5d shows that the prediction accuracy can be relatively improved by increasing the step time t_{step}.

To improve the reservoir performance further, we also proposed a new method for output reconstruction. Instead of using only the reservoir's output from the current step, we concatenate the response matrix A at each column with output states from previous steps to form a new matrix $A^* \in \mathbf{R}^{(N_r \cdot (1+n_p)) \times T_r}$, where n_p is the number of previous steps' states. In this way, the information of the reservoir states for training are enriched through compensation of previous states from reservoir. Better accuracy of prediction can then be achieved, as shown in Fig. S5e in the case $N_r = 50$. As a final illustration, Fig. S5f also shows selected training set (orange) and testing set (green dashed) for predictions for $H = 20$ and $n_p = 16$ (the red dot in Fig. S5e) compared to the true trajectory (blue) of Mackey-Glass time series, using the one-skyrmion system as reservoir, simulated by Mumax and the trained Neural ODE.

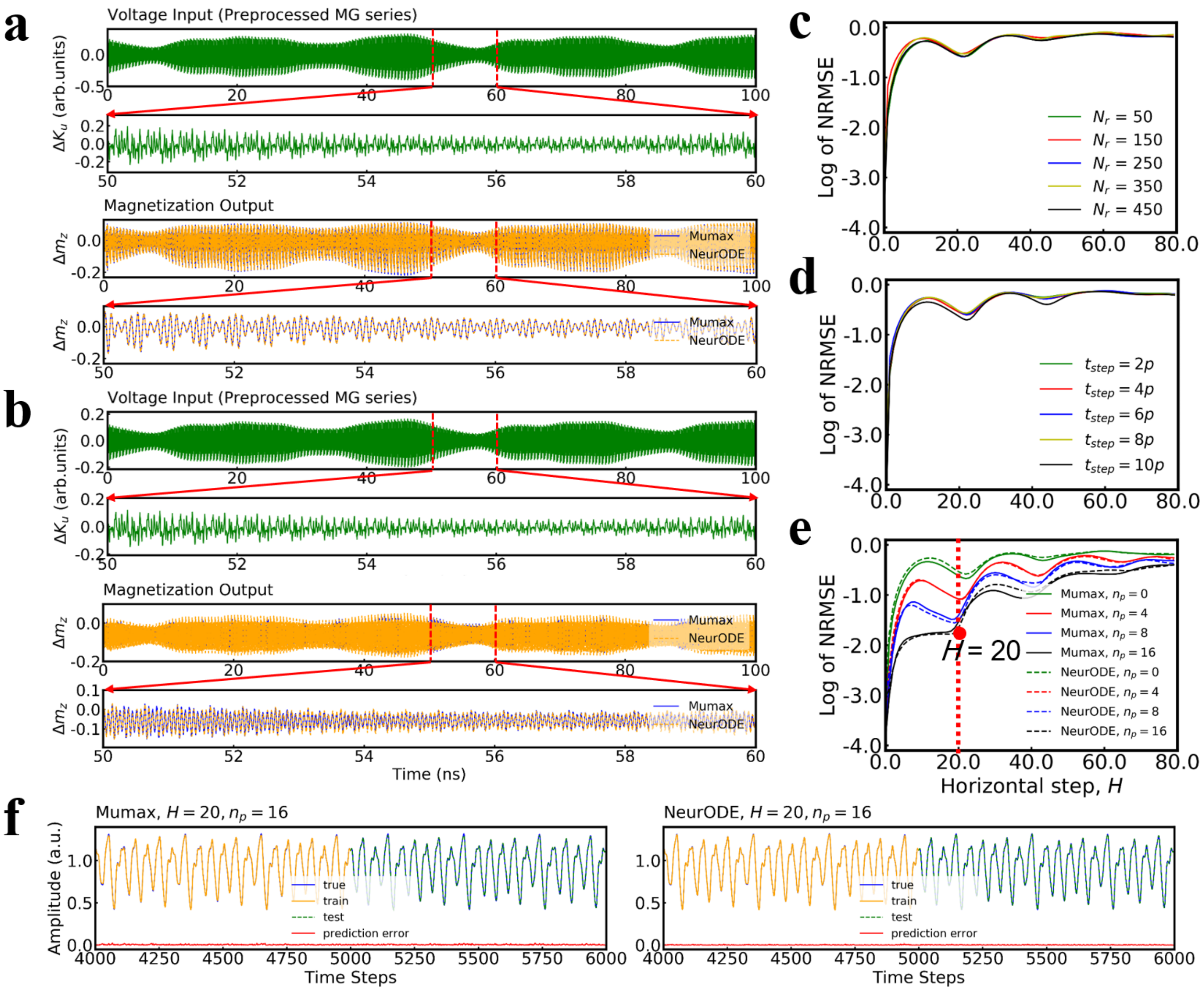

Supplementary Figure 5. Evaluation of the Mackey-Glass time-series prediction task. Selected reservoir output states, obtained continuously in time, computed by using Mumax and the trained Neural ODE, with preprocessed Mackey-Glass time series as input. The results are presented for the one-skyrmion system without grain inhomogeneity in **a** and for the multi-skyrmions system with grain inhomogeneity in **b**. Prediction accuracy in terms of NRMSE as a function of predicted horizontal steps for different **c** N_r values, **d** time durations t_{step} for each preprocessed input value fed into the reservoir, and **e** numbers of considered previous steps' states n_p. **f** Selected training set (orange) and testing set (green, dashed) for Mackey-Glass predictions for H=20 and $n_p = 16$ (red dot in Fig. S1**e**) compared to the true trajectory (blue) of Mackey-Glass time series using one skyrmion system as reservoir, simulated by Mumax and Neural ODE.

Supplementary Note 5: Modeling of the skyrmion system with electric current as input

The main article focuses on the voltage control of skyrmion-based devices. Skyrmions can also be moved by an electric current through current-induced spin–orbit torques (see Fig. S6a). Skyrmions are easily deformed when they are moving in a confined nanodisk, making this situation quite complex. NeurODE are highly attractive for modeling this situation, and we demonstrate here that the trajectory of a single skyrmion (position of the skyrmion core x_c, y_c) as well as the resulting mean magnetization of the device can be modeled using a Neural ODE.

The training dataset is again obtained from Mumax simulations. We use as input a random sine electric current, with amplitude of current density ranging from -1$\times 10^{11}$ to 1×10^{11} A/m^2, and a frequency $f = 0.5$ GHz (see Fig. S6c). The output trajectory of the skyrmion is recorded every $p = 20$ ps. A three-layer neural network with 100 units in each hidden layer is trained using 40,000 position data points. The final training output, as well as its comparison with the Mumax results, are shown in Fig. S6c. To further infer the magnetization information, another three-layer neural network M_θ^{out} with 50 units in each hidden layer was trained to recognize the variation of mean perpendicular magnetization Δm_z with skyrmion positions as input into the neural network (see Fig. S6b).

The trained Neural ODE is then evaluated on the task of Mackey-Glass time-series prediction, as in the voltage-control case. The preprocessed Mackey-Glass series, with $N_r = 50$, in the form of an electric current, is sent into Mumax and Neural ODE, correspondingly, with $t_{step} = 8p$. We obtained 410,000 data points (corresponding to 8,200 points of Mackey-Glass series) of final output trajectory after a simulation time of more than 40 days for Mumax and 80 minutes for Neural ODE. The corresponding magnetization Δm_z is then calculated by sending the output from Neural ODE into M_θ^{out}. MSE of Δm_z over the whole 410,000 data points computed by Neural ODE and M_θ^{out} is as low as 7.09×10^{-5} compared to that simulated by Mumax (see Fig. S7a).

The Mackey-Glass time series prediction error (NRMSE) as a function of predicted horizontal step by Mumax and Neural ODE is shown in Fig. S7b. Fig. S7c shows the selected comparative prediction results of $H = 1$ and $H = 25$ (red dots in b) obtained by Mumax and Neural ODE model respectively. Excellent agreement is again seen. Here, we used the first $T_r = 5,000$ points for training and the rest for testing.

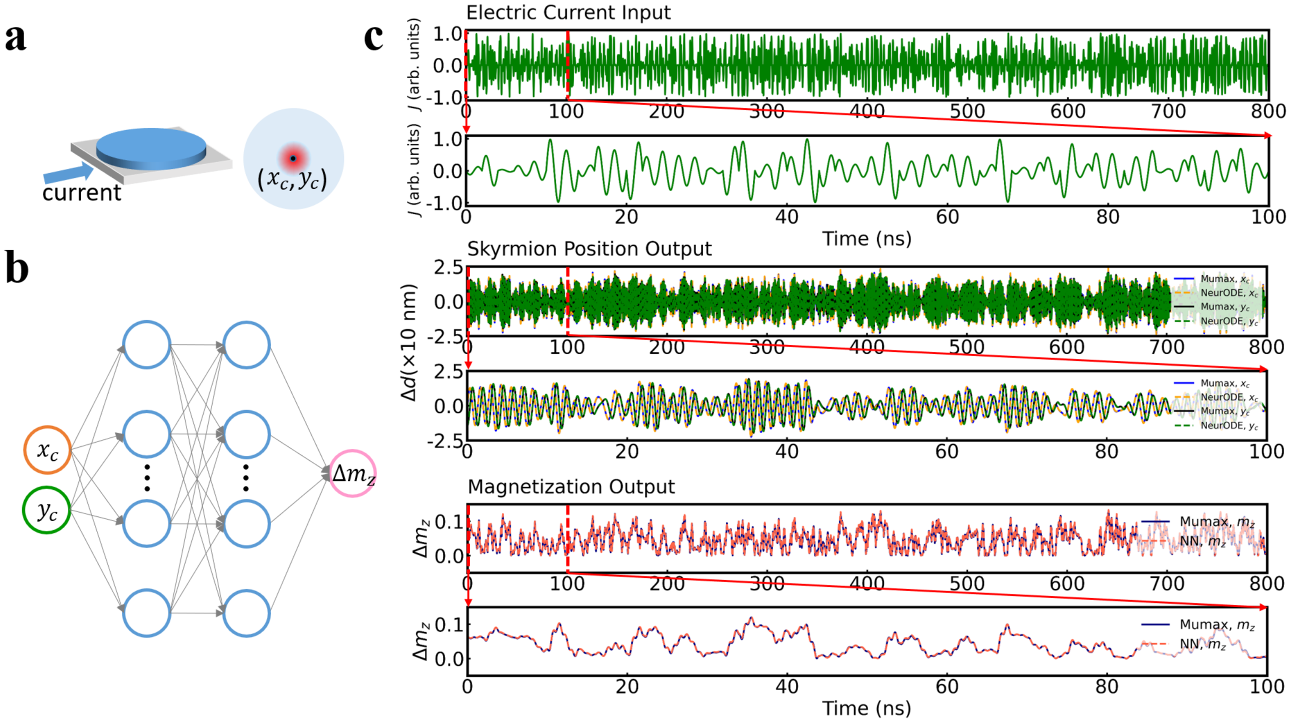

Supplementary Figure 6. **Modeling of the skyrmion system with electric current as input.** **a** Schematic of the nanodisk where skyrmion exists in the magnetic layer (blue), and electric current applied through the heavy metal layer (grey) is used as an external input. **b** Topology of the neural network used for obtaining the mean perpendicular magnetization Δm_z from the skyrmion core position (x_c, y_c). **c** Random sine electric current input, with amplitude ranging from -1$\times 10^{11}$ to 1×10^{11} A/m^2 and frequency $f = 0.5$ GHz, into the skyrmion system, and corresponding output trajectory of skyrmion position (x_c, y_c) calculated by Mumax and by the trained Neural ODE, as well as m_z, obtained from Neural ODE results and from Mumax.

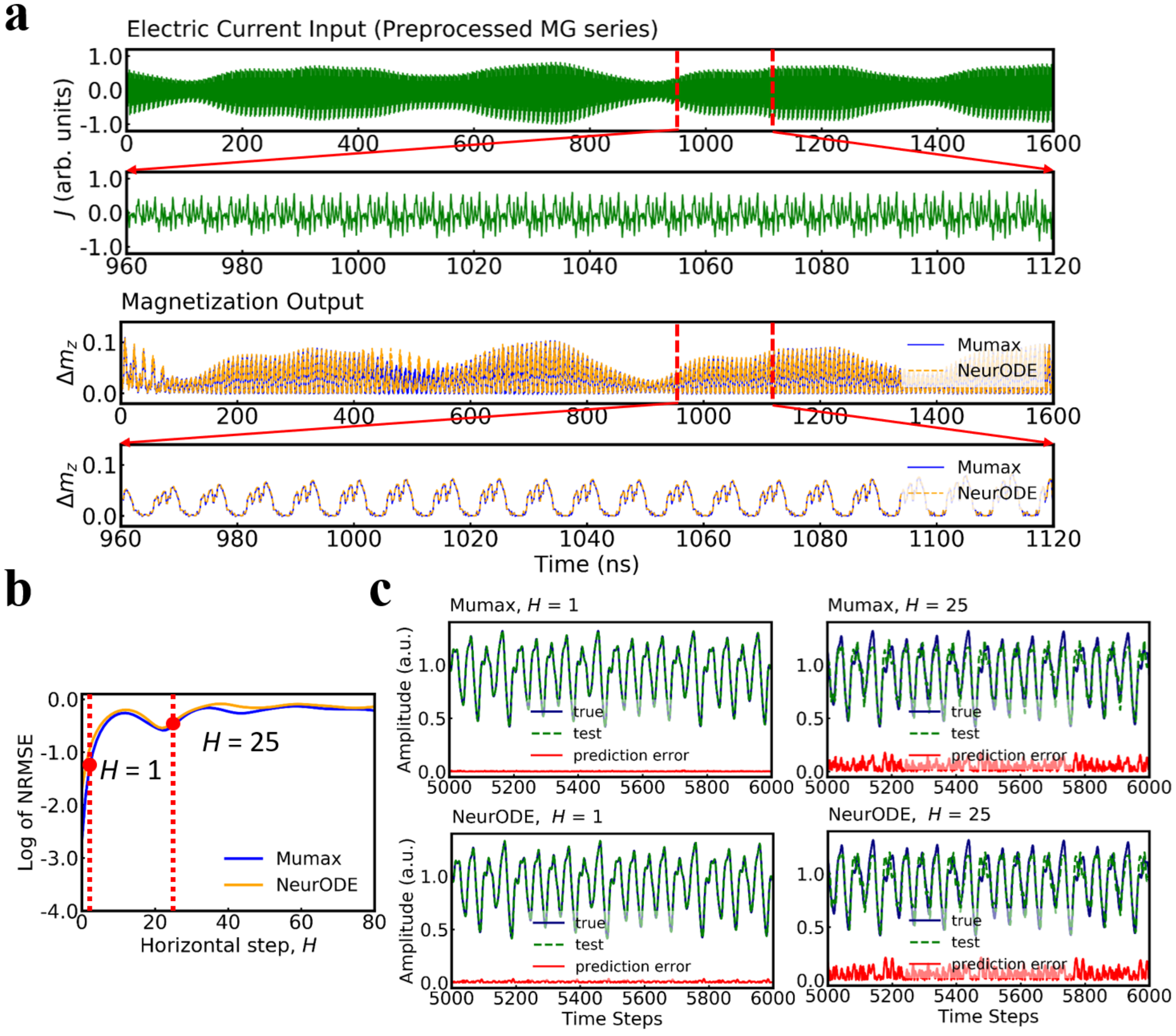

Supplementary Figure 7. Performance of Mackey-Glass time-series prediction, using Neural ODE and Mumax, for the skyrmion system with electric current input. a Selected reservoir output states, continuously in time, computed using Mumax and the trained Neural ODE with preprocessed Mackey-Glass time series as input. **b** Mackey-Glass time-series prediction error (NRMSE) as a function of predicted horizontal step by Mumax and Neural ODE. **c** Selected training set (orange) and testing set (green dashed) trajectories for Mackey-Glass predictions for $H = 1$ (short term prediction) and $H = 25$ (long term prediction) (red dots in **b**), compared to the true trajectory (blue) of Mackey-Glass time series, using the one-skyrmion system as reservoir by Mumax and Neural ODE.

Supplementary Note 6: Modeling abrupt switching and oscillations of a spin-valve nanopillar by Neural ODE

In this Note, we apply our method into modeling a situation, where significantly different behavior types may occur in the same system, depending on the input parameters. We have simulated a situation where a nanostructure may switch or generate sustained oscillations according to the value of the applied external magnetic field.

The training data is obtained from micromagnetic simulations of a spin-valve nanopillar, under the influence of a spin current polarized out-of-plane, and an out-of-plane magnetic field $H(t)$ applied in the z direction. The magnetization of the pinned layer is fixed along the negative z axis. The diameter and the thickness of the nanopillar are 80 nm and 2 nm, respectively. The key parameters used in the simulations are: saturation magnetization $M_s = 1.2$ MA/m, exchange stiffness $A = 20$ pJ/m, $K_u = 0.5$ MJ/m^3 and damping constant $\alpha = 0.1$. In the simulations, a constant electric direct current is applied with a current density of 10^{11}A/m^2. In the training dataset, artificial sinusoidal waveform of magnetic field with a variety of random amplitudes are applied sequentially as input, as shown below in Fig. S8a. In these artificial training conditions, the structures exhibits relatively complex and difficult-to-interpret behaviors, but which allow training a Neural ODE that is valid in all regimes.

For this purpose, we use a three-dimensional vector containing the information (m_x, m_y, m_z). A total length of 500-nanoseconds of dynamics is used to train the system. The trained results of m_z (dashed orange curve) and m_x (dashed olive curve), shown in Fig. S8a, demonstrate excellent agreement with the micromagnetic simulations.

In the test dataset, we apply a time-varying magnetic field at three different initial conditions of magnetization, covering a wide range of situations where the system is expected to exhibit either switching or sustained oscillations.

- Firstly, the magnetization is aligned in the positive z direction by applying an external field of $\mu_0 H$ = 0.65 T initially. A subsequent decreasing field is applied till a constant value to switch the magnetization (e.g., $\mu_0 H$ = -0.5 T) or to generate a sustained oscillation (e.g., $\mu_0 H$ = -0.1, 0.2, 0.3 T), as shown in the left column of Fig. S9a for different constant values.

- Secondly, the magnetization is aligned in the negative z direction by applying an external field of $\mu_0 H$ = -0.5 T initially, a subsequent decreasing field is applied till a constant value to switch the magnetization (e.g., $\mu_0 H$ = 0.7 T) or to generate a sustained oscillation (e.g., $\mu_0 H$ = 0.5, 0.2, -0.2 T), as shown in the middle column of Fig. S9a for different constant values.

- In the third scenario, no external field and only the constant electric current is applied initially. In this situation, the system exhibits self-sustained magnetization oscillations. A subsequent decreasing or increasing field is applied till a constant value to switch the magnetization (e.g., $\mu_0 H$ = -0.5, 0.7 T), as shown in the right column of Fig. S9a for different constant values.

We can see that in this test dataset, the results of Neural ODE and micromagnetic simulations are again in excellent agreement. The Neural ODE is able to predict all the various behaviors, equivalently to micromagnetic simulations.

For clearer comparisons, we further extract the oscillation frequency at different magnetic fields based on the results from Neural ODE and the micromagnetic simulations, as shown in Fig. S9b. This example demonstrates that the Neural ODE is able to describe different dynamical behaviors (switching and sustained oscillations) under different input parameters.

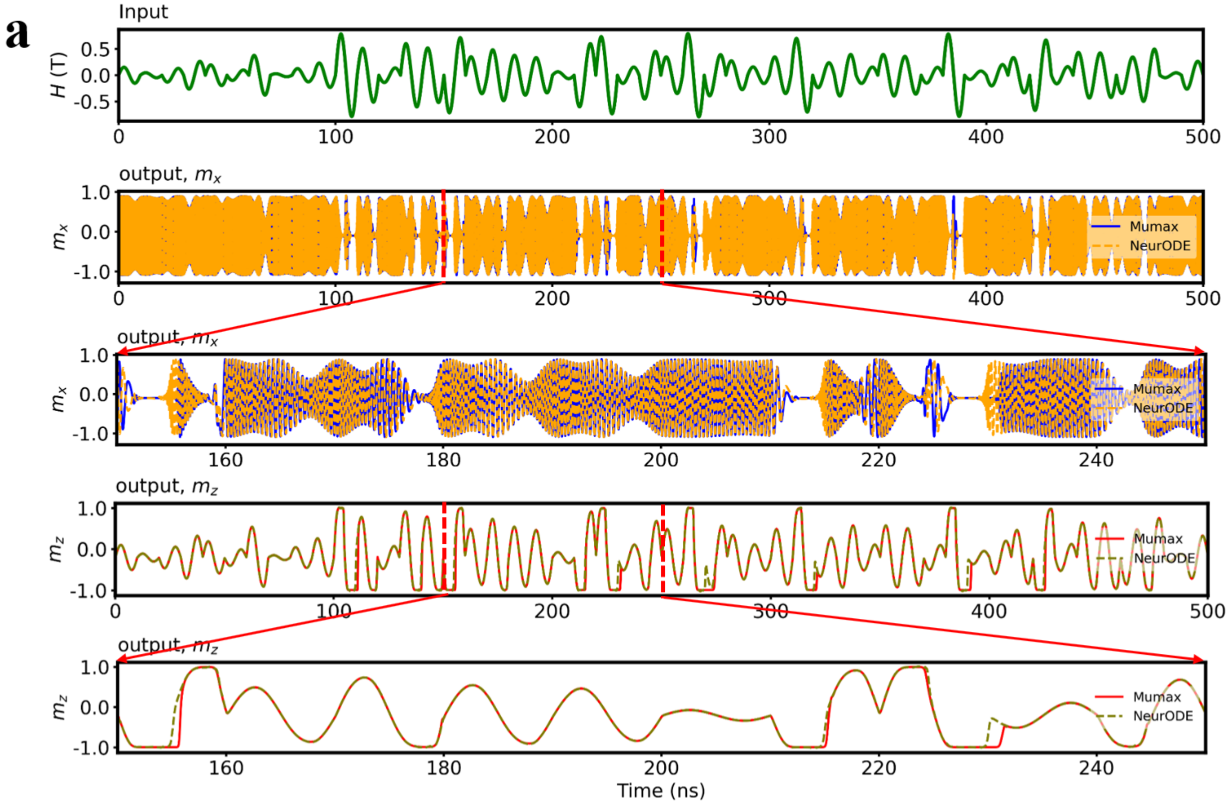

Supplementary Figure 8. Abrupt switching and oscillations of a spin-valve nanopillar modeled by Neural ODE (train dataset). a Input and output dynamics in the training dataset. The green curve shows the random sinusoidal input waveform of magnetic field with a variety of amplitudes applied in the z direction. The blue and red curves show the mean output magnetization of x component (m_x) and z component (m_z), respectively, obtained from micromagnetic simulations. The dashed orange (m_x) and dashed olive curves (m_x) are the corresponding trained results of Neural ODE.

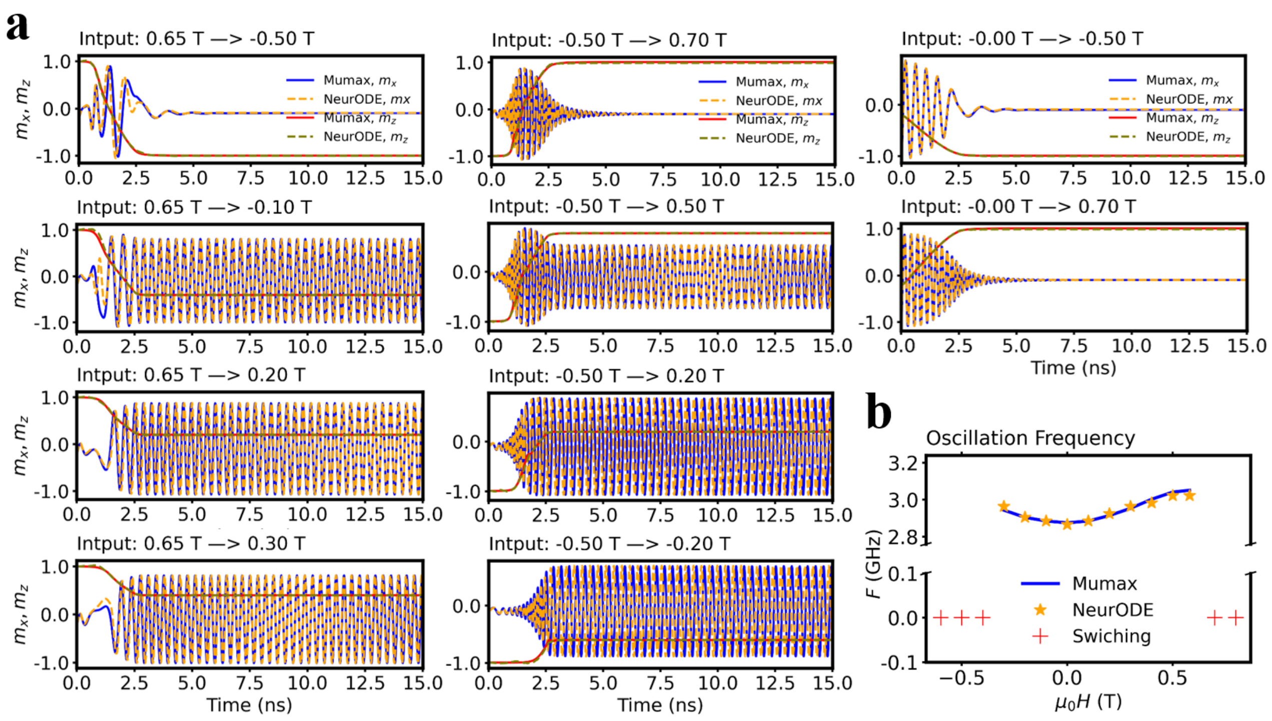

Supplementary Figure 9. **Abrupt switching and oscillations of a spin-valve nanopillar modeled by Neural ODE (test dataset).** **a** Neural ODE test results under different input parameters of the external magnetic field and at three different initial conditions of magnetization (from left to right). In the left column, the magnetization is aligned in the positive z direction by applying an external field of $\mu_0 H$ = 0.65 T initially, a subsequent decreasing field is applied till a constant value to switch the magnetization ($\mu_0 H$ = -0.5 T) or to generate a sustained oscillation ($\mu_0 H$ = -0.1, 0.2, 0.3 T). In the middle column, the magnetization is aligned in the negative z direction by applying an external field of $\mu_0 H$ = -0.5 T initially, a subsequent increasing field is applied till a constant value to switch the magnetization ($\mu_0 H$ = 0.7 T) or to generate a sustained oscillation ($\mu_0 H$ = 0.5, 0.2, -0.2 T). In the right column, no external field and only the constant electric current is applied initially, the system exhibits self-sustained magnetization oscillations. A subsequent decreasing or increasing field is applied till a constant value to switch the magnetization ($\mu_0 H$ = -0.5, 0.7 T). The blue and red curves show the mean output magnetization of x component (m_x) and z component (m_z), respectively, from micromagnetic simulations. The dashed orange (m_x) and dashed olive curves (m_x) are the corresponding trained results of Neural ODE. **b** Oscillation frequency F at different magnetic fields obtained by using the results from Neural ODE (orange star) and the micromagnetic simulations (blue curve). The red cross symbols represent the switching behavior at the specific amplitude of magnetic fields obtained by both Neural ODE and the micromagnetic simulations.

Supplementary Note 7: Spoken digit recognition performance of Neural ODE based on the experimental measurement of spintronic oscillator

This note provides additional results on the use of Neural ODEs to predict experimental measurements, on the spoken digit recognition task with spintronic oscillator. Fig. S10 presents the same results as Fig. 4 in the main body text for the spectrogram method (Fig. 4 in the main body text was presented with the cochlear method, see Methods section). Overall, the results appear highly similar to the cochlear method case. A Neural ODE is able to fit the training set very accurately if its dimension is higher than two (Figs. S10a-b). The trained neural ODE is able to predict the output signals of unseen experiments precisely (Fig. S10c). Finally, with the addition of noise (Fig. S10d), a Neural ODE is able to predict the accuracy of the experiment of the task of spoken digit recognition perfectly (Fig. S10d).

A surprising result is that, with the addition of noise, the spoken digit task performance degrades for the spectrogram method, while the task performance improved for the cochlear method (see Fig. 4, main body text). These results suggest that the output states from the reservoir are highly over-fitted in the noiseless cochlear case; noise suppresses this over-fitting effect. Conversely, in the spectrogram case, the reservoir computer operates in a non-over-fitted regime; noise then plays a more conventional detrimental role. The capability of Neural ODEs to capture such subtle behaviors showcases their impressive predictive power.

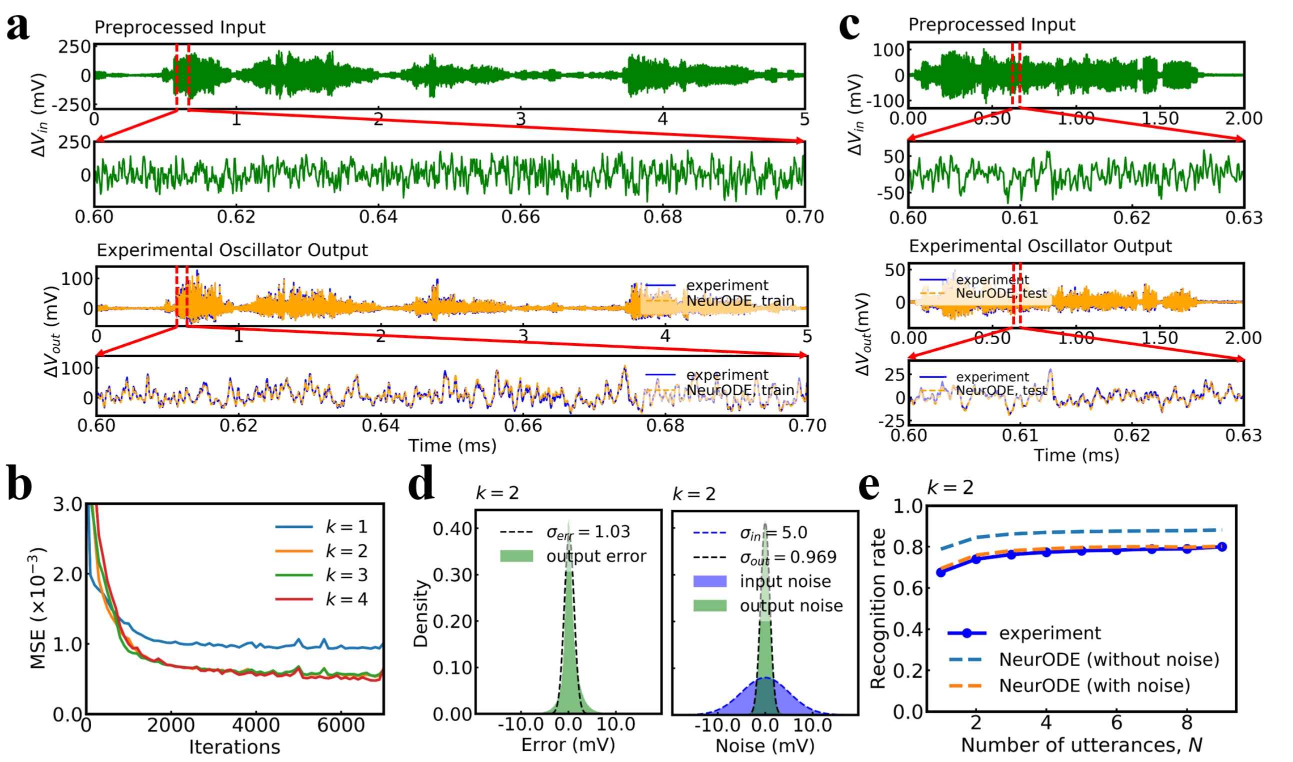

Supplementary Figure 10. Prediction of experimental results using Neural ODEs (spectrogram method). **a** Training output trajectory of voltage ΔV_{out} by Neural ODE (dashed orange) with corresponding preprocessed input ΔV_{in}, in comparison with the experimental measurement (blue), for $k = 2$. This training set is adopted from the first utterance of the first speaker. A three-layer neural network f_θ with each hidden layer of 100 units is trained. **b** Training loss (MSE) of Neural ODE with $k = 1,2,3,4$ as a function of iterations. **c** Selected response output trajectories predicted by the trained Neural ODE with corresponding preprocessed input, in comparison with the experimental output, for digit nine of the seventh utterance of the fifth speaker. **d** Left to right: Error distribution (green shadow) and fitted Gaussian distribution (dashed curve) extracted by computing the difference between the output predicted by Neural ODE and the experimental measurement, a Gaussian noise (purple shadow) added in the preprocessed input into the Neural ODE and the corresponding noise distribution (green shadow) with fitted pdf (black dashed curve) in the predicted output trajectory solved by Neural ODE. σ_{in} was adjusted so that $\sigma_{out} = 1.03$ mV is close to $\sigma_{err} = 0.97$ mV. **e** Spoken digit recognition rate in the testing set as a function of utterances N used for training. The solid curve, blue dashed curve, orange dashed curve are the experimental result, Neural ODE result with noise considered, Neural ODE result without any noise, respectively.

Supplementary Note 8: Mathematical derivations

A Converting a system of ODEs into a single higher-order ODE in one variable

Motivations Our idea originates in the insight that it is possible to convert a set of the first-order differential equations in multiple variables into a single higher-order differential equation in one variable. For example, let us consider the case with a single hidden variable $\tilde{y}_2$,

$$\begin{bmatrix} \dot{y}_1 \\ \dot{\tilde{y}}_2 \end{bmatrix} = \begin{bmatrix} ay_1 + b\tilde{y}_2 \\ cy_1 + d\tilde{y}_2 \end{bmatrix}. \tag{1}$$

By substituting $\tilde{y}_2$ and its derivative $\dot{\tilde{y}}_2$ calculated from the first ODE into the second one, a second-order ODE with variable y_1 can be derived as $\ddot{y}_1 = (a+d)\dot{y}_1 + (bc-ad)y_1$, where $\tilde{y}_2$ no longer appears. This equation is equivalent to the following ODE in terms of y_1 and $y_2 = \dot{y}_1$

$$\begin{bmatrix} \dot{y}_1 \\ \dot{y}_2 \end{bmatrix} = \begin{bmatrix} y_2 \\ (a+d)y_2 + (bc-ad)y_1 \end{bmatrix}. \tag{2}$$

This simple derivation suggests that an appropriate way for training a Neural ODE of m internal variables where only one variable y_1 is accessible is to train a Neural ODE where the state vector $\mathbf{y}$ is composed of y_1 and its $(m-1)^{\text{th}}$-order derivatives.

Theorem 1 (for linear system) Let A be an $n \times n$ matrix, and let $p_A(\lambda)$ be the characteristic polynomial of A. Let $\mathbf{y}(t) = (y_1(t), \ldots, y_n(t))^T$ be a solution to the system of ODEs

$$\dot{\mathbf{y}} = A\mathbf{y}. \tag{3}$$

Then, each coordinate function $y_j(t)$ satisfies the nth-order differential equation

$$p_A(D)y_j = 0. \tag{4}$$

Proof: Rewrite the differential equation using the operator notation as

$$D\mathbf{y} = A\mathbf{y}, \tag{5}$$

where $D\mathbf{y}$ indicates differentiation of the vector $\mathbf{y}$ by $\frac{d}{dt}$ and $A\mathbf{y}$ indicates multiplication of vector $\mathbf{y}$ by the matrix A. By differentiating both sides of the ODEs, we obtain

$$\begin{aligned} D^2\mathbf{y} &= D(A\mathbf{y}) = A(D\mathbf{y}) = A^2\mathbf{y} \\ D^3\mathbf{y} &= A^3\mathbf{y} \\ &\vdots \\ D^n\mathbf{y} &= A^n\mathbf{y}. \end{aligned} \tag{6}$$

Hence, $p(D)\mathbf{y} = p(A)\mathbf{y}$ for any polynomial $p(\lambda)$ by linearity. The Cayley-Hamilton theorem states that $p_A(A) = 0$. Therefore, $p_A(D)\mathbf{y} = 0$. So, for each coordinates, $p_A(D)y_j = 0$.

Differential elimination procedure (for nonlinear system). The procedures of converting systems of ODEs to a single-higher order ODE in one variable was mathematically proved for nonlinear systems, we refer readers to

refs[2–4] for further details. Here, we are giving the general ideas of the procedures followed in these references. We consider a system of nonlinear ODEs of the form ($\dot{\mathbf{x}} = f(\mathbf{x})$):

$$\begin{aligned} \dot{x}_1 &= f_1(x_1, x_2, \ldots, x_n) \\ \dot{x}_2 &= f_2(x_1, x_2, \ldots, x_n) \\ &\vdots \\ \dot{x}_n &= f_n(x_1, x_2, \ldots, x_n) \\ y &= x_j, j \in (1, \ldots, n). \end{aligned} \tag{7}$$

Here $\mathbf{x} = (x_1, x_2, \ldots, x_n)$ is an n-dimensional state variable, and y is considered as an output vector, which could be any component of the state variable. We assume that $f(\mathbf{x})$ is rational polynomial functions of the state variables, a reasonable assumption in most applications. Our aim is to obtain a single ODE in the variable y.

The procedure can be implemented by taking a sufficient number of derivatives of the system, followed by computation of a Gröbner Basis of the new system[2–4]. In general, for elimination to work, the number of equations must be strictly greater than the number of unknowns[2,3]. Firstly, from the output equation, we can obtain $\dot{y} = \dot{x}_j$ and $\ddot{y} = \ddot{x}_j$. This, however, introduces the second derivative of x_j, and thus differentiation of the corresponding state variable equation is needed:

$$\begin{aligned} \dot{x}_1 &= f_1(x_1, x_2, \ldots, x_n) \\ \dot{x}_2 &= f_2(x_1, x_2, \ldots, x_n) \\ &\vdots \\ \dot{x}_n &= f_n(x_1, x_2, \ldots, x_n) \\ y &= x_j \\ \dot{y} &= \dot{x}_j \\ \ddot{y} &= \ddot{x}_j \\ \ddot{x}_j &= \sum_{i=1}^{n} \frac{\partial f_i}{\partial x_i} \dot{x}_i, j \in (1, \ldots, n), \end{aligned} \tag{8}$$

Now, at the second step, we have $n+4$ equations and $2n+1$ unknowns $(x_1, x_2, \ldots, x_n, \dot{x}_1, \dot{x}_2, \ldots, \dot{x}_n, \ddot{x}_j)$. So, for $n > 2$, the number of unknowns is greater than or equal to the number of equations. Next, at step three, we take an additional derivative of the output equation, which causes the third derivative of the state variable to appear. Thus, we take the corresponding derivative of the state variable x_j and add the following equations to the system:

$$\begin{aligned} \dddot{y} &= \dddot{x}_j \\ \dddot{x}_j &= \sum_{i=1}^{n} \left[\frac{\partial^2 f_j}{\partial x_i \partial t} \dot{x}_i + \frac{\partial f_j}{\partial x_i} \ddot{x}_i\right], j \in (1, \ldots, n) \\ \ddot{x}_k &= \sum_{i=1}^{n} \frac{\partial f_k}{\partial x_i} \dot{x}_i, \text{ for } k = 1, \ldots, n \text{ and } k \neq j. \end{aligned} \tag{9}$$

In doing this, we add $2+n-1 = n+1$ equations and $1+(n-1) = n$ unknowns; so, we have $2n+5$ equations and $3n+1$ unknowns. So, for $n > 3$, the number of unknowns is greater than or equal to the number of equations. Differentiation of the output equation and corresponding state variable equations is continued until the number of equations is greater than the number of unknowns.

Lemma 1 Let the system consist of n state variables. There is always an equal number of equations and unknowns when there are $n-1$ derivatives of the output equation.

Proof: Initially, there are $n+1$ equations and $2n$ unknowns. After one step derivative on the output, there are always $n+2$ equations and $2n$ unknowns. Thus, there are $n-2$ more unknowns than equations. In each step thereafter, we add one more equation than unknown, so at the $(n-1)$th step ($(n-1)$th-order derivatives to the output), there are zero more unknowns than equations. Thus, the Lemma is proven.

Corollary to Lemma 1 At the n-th step, there is one more equation than unknown.

This suggests that an ODE of the same (or possibly lower) differential order as the number of state variables can always be obtained. In other words, since there is one more equation than unknown at step n, an ODE of differential order n, or lower, can be obtained.

Remark: If there is a time-dependent input e added into the system, we use the same elimination procedure. Then, after taking the second derivative of the output y, we have the following $n+4$ equations

$$\begin{aligned}
\dot{x}_1 &= f_1(x_1, x_2, \dots, x_n, e) \\
\dot{x}_2 &= f_2(x_1, x_2, \dots, x_n, e) \\
&\vdots \\
\dot{x}_n &= f_n(x_1, x_2, \dots, x_n, e) \\
y &= x_j \\
\dot{y} &= \dot{x}_j \\
\ddot{y} &= \ddot{x}_j \\
\ddot{x}_j &= \sum_{i=1}^{n} \frac{\partial f_i}{\partial x_i} \dot{x}_i + \frac{\partial f_j}{\partial e} \dot{e}, j \in (1, \dots, n),
\end{aligned} \tag{10}$$

in which the first derivative of e is included. Thus, after taking nth-order derivatives to the output y at n-th step, the first to $(n-1)$th-order derivative of e are included, and a single nth-order ODE in variable y can be obtained, which explains why the time-delayed state of input $e(t)$ should be included when training the neural network.

B Noise level for higher-order derivatives

We now suppose the observed data $s(t) = y(t) + n(t)$ is a stationary random process[5], recorded with measurement noise $n(t)$, identically distributed with variance σ_{noi}^2, zero mean, and a normalized autocorrelation function $c_{noi}(\tau) = E[n(t)n(t-\tau)]/\sigma_{noi}^2$. Let $y(t)$ be the clean variable with variance $\sigma_{sig}^2 = E[y(t)^2] - E[y(t)]^2$ and normalized autocorrelation function $c_{sig}(\tau) = E[(y(t) - E[y(t)])(y(t-\tau) - E[y(t)])]/\sigma_{sig}^2$. We assume that the data is recorded with a high sampling rate Δt, such that the successive observations are strongly correlated. If we take the first derivative through the following form

$$\dot{s}(t) = \frac{1}{2\Delta t}(s(t+\Delta t) - s(t-\Delta t)) \tag{11}$$

The real signal derivative is, neglecting terms higher than the second order in Δt,

$$\dot{y}(t) = \frac{1}{2\Delta t}(y(t+\Delta t) - y(t-\Delta t)) \tag{12}$$

Therefore the corresponding (derivative) signal variance is

$$\begin{aligned}
\sigma^2_{sig,der} &= E[\dot{y}(t)^2] - E[\dot{y}(t)]^2 \\
&= E[\dot{y}(t)^2] \\
&= E[\frac{1}{4\Delta t^2}(y(t+\Delta t) - y(t-\Delta t))^2] \\
&= \frac{1}{2\Delta t^2}(E[y(t)^2] - E[y(t-\Delta t)y(t+\Delta t)]) \\
&= \frac{1}{2\Delta t^2}(1 - c_{sig}(2\Delta t))
\end{aligned} \tag{13}$$

The corresponding noise of the derivative signal $\dot{s}(t) - \dot{y}(t)$ is

$$\sigma^2_{noi,der} = \frac{1}{2\Delta t^2}(1 - c_{noi}(2\Delta t)) \tag{14}$$

Thus, the noise to signal ratio of the second derivative is

$$\frac{\sigma_{noi,der}}{\sigma_{sig,der}} = \frac{\sigma_{noi}}{\sigma_{sig}}\sqrt{\frac{1 - c_{noi}(2\Delta t)}{1 - c_{sig}(2\Delta t)}}. \tag{15}$$

For white noise, the autocorrelation function is an impulse at lag 0, thus $c_{noi}(2\Delta t)$ is close to zero. Therefore, the noise-to-signal ratio of the derivative ($\sigma_{noi,der}/\sigma_{sig,der}$) can be much larger than the original signal ($\sigma_{noi}/\sigma_{sig}$) if the sampled adjacent signal is strongly correlated, which is a common situation in most applications. This consideration explains our choice, in the main paper, to use delayed signals instead of derivates as state variables for Neural ODEs for systems with incomplete information of dynamics.

Supplementary Note 7: Alternative interpretation based on the embedding theorem

An alternative interpretation for our method can also be obtained through the "embedding theorem" for state-space reconstruction of dynamical systems. It provides additional insight into our technique. Let us assume the system can be described by an ensemble of physical variables represented by vector $\mathbf{u}$, and that the variable that can we can measure (e.g., a voltage) can be computed by a measurement function $s(\mathbf{u})$. Let us assume that the underlying ODE describing the physics of the system is $\dot{\mathbf{u}} = F(\mathbf{u},t)$. Using the point of view of the embedding theorem, our technique corresponds to changing the physical variables $\mathbf{u}$ to new variables $\mathbf{y}$. We have $y_1 = s(\mathbf{u}(t))$ and $y_2 = y_1(t+\Delta t_d) = s(\mathbf{u}(t) + \int_t^{t+\Delta t_d} F(\mathbf{u},t')\,dt')$. We define the transformation G by $G(\mathbf{u}(t)) = \mathbf{u}(t) + \int_t^{t+\Delta t_d} F(\mathbf{u},t')\,dt'$ so that we have $y_2 = s(G(\mathbf{u}(t))$ and, for all i values,

$$y_i = y_1(t + i\Delta t_d) = s(G^i(\mathbf{u}(t))). \tag{16}$$

According to the embedding theorem[5–8], for almost every smooth measurement function s and positive Δt_d value, such a delay map $\mathbf{y}(t)$ is a smooth, one-to-one coordinate transformation with a smooth inverse if its dimension k is at least twice the capacity dimension of the dynamical system (also called fractal dimension or box-counting dimension). This consideration provides an alternative justification for the choice of these coordinates, in the main paper.

References

1. Casdagli, M., Eubank, S., Farmer, J. D. & Gibson, J. State space reconstruction in the presence of noise. *Phys. D: Nonlinear Phenom.* **51**, 52–98 (1991).
2. Meshkat, N., Anderson, C. & DiStefano III, J. J. Alternative to ritt's pseudodivision for finding the input-output equations of multi-output models. *Math. biosciences* **239**, 117–123 (2012).
3. Jirstrand, M. *Algebraic methods for modeling and design in control* (Division of Automatic Control, Department of Electrical Engineering . . . , 1996).
4. Forsman, K. *Constructive commutative algebra in nonlinear control theory*. Ph.D. thesis, Linköping University (1991).
5. Kantz, H. & Schreiber, T. *Nonlinear time series analysis*, vol. 7 (Cambridge university press, 2004).
6. Whitney, H. Differentiable manifolds. *Annals Math.* 645–680 (1936).
7. Takens, F. Detecting strange attractors in turbulence. In *Dynamical systems and turbulence, Warwick 1980*, 366–381 (Springer, 1981).
8. Sauer, T., Yorke, J. A. & Casdagli, M. Embedology. *J. statistical Phys.* **65**, 579–616 (1991).
